# Ethyl­enediammonium tetra­aqua­disulfato­cadmate

**DOI:** 10.1107/S1600536811030005

**Published:** 2011-08-02

**Authors:** Walid Rekik, Houcine Naïli, Tahar Mhiri, Thierry Bataille

**Affiliations:** a Laboratoire de l’État Solide, Département de Chimie, Faculté des Sciences de Sfax, Université de Sfax, BP 1171, 3000 Sfax, Tunisia; b Laboratoire de Chimie du Solide et Inorganique Moléculaire (CNRS, UMR 6511), Université de Rennes I, Avenue du Général Leclerc, 35042 Rennes Cedex, France

## Abstract

The crystal structure of the title compound, [NH_3_(CH_2_)_2_NH_3_][Cd(SO_4_)_2_(H_2_O)_4_], consists of [Cd(SO_4_)_2_(H_2_O)_4_]^2−^ anions that are built from octa­hedral Cd(H_2_O)_4_O_2_ and SO_4_ tetra­hedral units linked by corner sharing. The ethyl­ene­diamminium cations are linked to the anions *via* N—H⋯O hydrogen bonds. The asymmetric unit contains one-half of the compound, the other half being related to the first by an inversion centre. The crystal structure presents alternate stacking of the inorganic and organic layers along the crystallographic *b* axis. The structure cohesion and stability is further assured by O(water)—H⋯O hydrogen bonds.

## Related literature

For our previous work on the synthesis, characterization and properties of mixed metal sulfates and amines, see: Rekik *et al.* (2005 ▶, 2007 ▶, 2008 ▶, 2009*a* ▶); Naïli *et al.* (2006 ▶); Yahyaoui *et al.* (2007 ▶). For the manganese, iron, cobalt and magnesium analogs of the title compound, see: Chaabouni *et al.* (1996 ▶); Held (2003 ▶); Rekik *et al.* (2008 ▶, 2009*b* ▶). For our previous work on the synthesis, characterization and properties of mixed metal sulfates and amines, see: Rekik, Naïli, Bataille & Mhiri (2006 ▶); Rekik, Naïli, Bataille, Roisnel & Mhiri (2006 ▶).
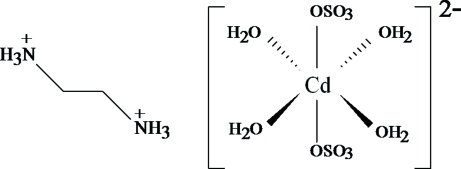


## Experimental

### 

#### Crystal data

(C_2_H_10_N_2_)[Cd(SO_4_)_2_(H_2_O)_4_]
*M* *_r_* = 438.70
Triclinic, 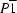

*a* = 6.9114 (2) Å
*b* = 7.3056 (2) Å
*c* = 7.3629 (1) Å
α = 74.013 (2)°
β = 71.731 (1)°
γ = 78.043 (1)°
*V* = 336.39 (1) Å^3^
*Z* = 1
Mo *K*α radiation
μ = 1.99 mm^−1^
*T* = 293 K
0.12 × 0.11 × 0.07 mm

#### Data collection

Nonius KappaCCD diffractometer
Absorption correction: analytical (de Meulenaer & Tompa, 1965 ▶) *T*_min_ = 0.817, *T*_max_ = 0.885
8732 measured reflections
4684 independent reflections
4352 reflections with *I* > 2σ(*I*)
*R*_int_ = 0.040

#### Refinement

*R*[*F* ^2^ > 2σ(*F* ^2^)] = 0.030
*wR*(*F* ^2^) = 0.070
*S* = 1.04
4684 reflections
105 parameters
6 restraints
H atoms treated by a mixture of independent and constrained refinement
Δρ_max_ = 0.90 e Å^−3^
Δρ_min_ = −1.49 e Å^−3^

### 

Data collection: *COLLECT* (Nonius, 1998 ▶); cell refinement: *SCALEPACK* (Otwinowski & Minor, 1997 ▶); data reduction: *DENZO* (Otwinowski & Minor, 1997 ▶) and *SCALEPACK*; program(s) used to solve structure: *SHELXS97* (Sheldrick, 2008 ▶); program(s) used to refine structure: *SHELXL97* (Sheldrick, 2008 ▶); molecular graphics: *DIAMOND* (Brandenburg & Berndt, 1999 ▶); software used to prepare material for publication: *WinGX* (Farrugia, 1999 ▶).

## Supplementary Material

Crystal structure: contains datablock(s) global, I. DOI: 10.1107/S1600536811030005/go2021sup1.cif

Structure factors: contains datablock(s) I. DOI: 10.1107/S1600536811030005/go2021Isup2.hkl

Additional supplementary materials:  crystallographic information; 3D view; checkCIF report

## Figures and Tables

**Table 1:** Selected bond lengths (Å)

Cd—O*W*1	2.2511 (12)
Cd—O4	2.2789 (9)
Cd—O*W*2	2.2887 (10)

**Table 2:** Hydrogen-bond geometry (Å, °)

*D*—H⋯*A*	*D*—H	H⋯*A*	*D*⋯*A*	*D*—H⋯*A*
N—H0*A*⋯O4	0.89	1.93	2.8155 (15)	177
N—H0*B*⋯O2^ii^	0.89	1.99	2.8378 (16)	160
N—H0*C*⋯O3^iii^	0.89	2.03	2.8767 (15)	160
O*W*1—H11⋯O2^iv^	0.83 (2)	1.90 (2)	2.7342 (15)	176 (3)
O*W*1—H12⋯O3^v^	0.85 (2)	1.89 (2)	2.7293 (17)	173 (3)
O*W*2—H21⋯O1^vi^	0.83 (2)	2.01 (2)	2.8177 (14)	163 (2)
O*W*2—H22⋯O1^vii^	0.84 (2)	1.89 (2)	2.7150 (15)	165 (2)

Symmetry codes: (ii) 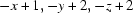; (iii) 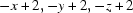; (iv) 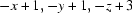; (v) 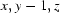; (vi) 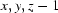; (vii) 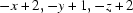.

**Fig. 1.**
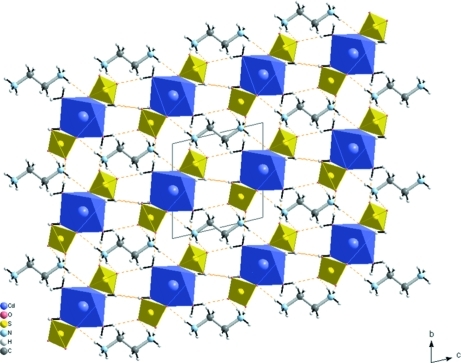
Projection of the crystal structure of the title compound along the a axis, with hydrogen bonds indicated as dashed lines.

**Fig. 2.**
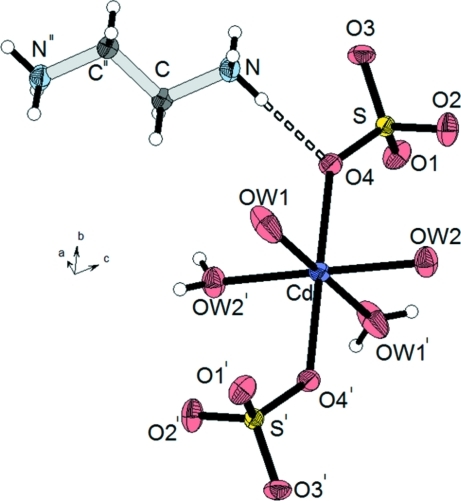
A part of the crystal structure of the title compound showing the asymmetric unit (expanded by symmetry to give complete organic cation and trimeric unit) and atom numbering. Displacement ellipsoids are drawn at the 50% probability level. Hydrogen bonds are represented by dashed lines.[Symmetry codes: (I) -x + 1, -y + 1, -z + 2; (II) -x + 2, -y + 2, -z + 1.]

## supplementary crystallographic information

### Comment

The chemistry of organic-inorganic hybrid materials has received increasing attention over the last few years, mainly with the idea of using amines as templates and associating transition metals. A great interest has been shown in some organically templated metal sulfate because of their open-framework structure and ferroelastic and ferroelectric propreties. We note also that a previous work of synthesis and characterization of mixed metal sulfates and amines, leading to many important physical properties, has been realized in our laboratory (Rekik *et al.*, 2005; Naïli, *et al.*, 2006; Rekik *et al.*, 2007; Yahyaoui *et al.*, 2007; Rekik *et al.*, 2008; Rekik *et al.*, 2009*a*). In the course of our investigations on new sulfate materials having interesting properties, we report here the chemical preparation and the structural characterization of a new ethylenediammonium cadmium (II) teraaquadisulfato, [NH_3_(CH_2_)_2_NH_3_][Cd(SO_4_)_2_(H_2_O)_4_]. The title compound is isostructural with the manganese, iron, cobalt and magnesium related phases (Chaabouni *et al.*, 1996; Held, 2003; Rekik *et al.*, 2008; Rekik *et al.*, 2009*b*). As it can be seen in figure 1, the crystal structure shows an alternate stacking of inorganic layers of tetraaquabis(sulfato-O)cadmium anions, [Cd(SO_4_)_2_(H_2_O)_4_]^2-^, and organic layers of [NH_3_(CH_2_)_2_NH_3_]^2+^ cations along the crystallographic *b* axis. Anions and cations are linked together through N—H···O hydrogen bonds to form a three-dimensional network. The asymmetric unit (Fig. 2) of the title compound contains only one cadmium atom located at a symmetry centre, only one sulfate tetrahedron and ethylendiammonium cation lying about inversion centre. The Cd(II) central atom is octahedrally coordinated by one oxygen atom of sulfate group, two water molecules and the corresponding centrosymmetrically located atoms. Each octahedron around Cd shares two oxygen atoms with two sulfate groups to form trimeric units. These latest are stabilized and linked *via* OW—H···O hydrogen bonds giving rise to a three-dimensional inorganic framework delimiting tunnels along the three crystallographic axes. The negative charge of the inorganic part is compensated by ethylediammonium cations which are located on inversion centres in the inorganic framework cavities. The structure cohesion and stability are assured by two types of hydrogen bonds, OW—H···O and N—H···O.

### Experimental

Single-crystals of the title compound were grown by slow evaporation at room temperature of an aqueous solution of CdSO_4_.8(H_2_O)/C_2_H_8_N_2_ /H_2_SO_4_in a ratio 1:1:1. The product was filtered off and washed with a small amount of distilled water.

### Refinement

The aqua H atoms were located in difference map and refined with O—H distance restraints of 0.85 (2) Å and H—H distance restraints of 1.35 (2) Å. H atoms bonded to C and N atomswere positioned geometrically and allowed to ride on their parent atom, with C—H = 0.97 Å, N—H = 0.89 Å and *U*_iso_ = 1.2*U*_eq_(C, N). The 1 1 3 reflection has been omitted, (Iobs/Ical)/sigma greater than 10.

### Crystal data

**Table:**

(C_2_H_10_N_2_)[Cd(SO_4_)_2_(H_2_O)_4_]	*Z* = 1
*M**_r_* = 438.70	*F*(000) = 220
Triclinic, *P*1	*D*_x_ = 2.166 Mg m^−^^3^
Hall symbol: -P 1	Mo *K*α radiation, λ = 0.71073 Å
*a* = 6.9114 (2) Å	Cell parameters from 8732 reflections
*b* = 7.3056 (2) Å	θ = 2.9–42.2°
*c* = 7.3629 (1) Å	µ = 1.99 mm^−^^1^
α = 74.013 (2)°	*T* = 293 K
β = 71.731 (1)°	Prism, colourless
γ = 78.043 (1)°	0.12 × 0.11 × 0.07 mm
*V* = 336.39 (1) Å^3^	

### Data collection

**Table:**

Nonius KappaCCD diffractometer	4684 independent reflections
Radiation source: fine-focus sealed tube	4352 reflections with *I* > 2σ(*I*)
horizontally mounted graphite crystal	*R*_int_ = 0.040
Detector resolution: 9 pixels mm^-1^	θ_max_ = 42.2°, θ_min_ = 2.9°
CCD rotation images, thick slices scans	*h* = −13→13
Absorption correction: analytical (de Meulenaer & Tompa, 1965)	*k* = −9→13
*T*_min_ = 0.817, *T*_max_ = 0.885	*l* = −9→13
8732 measured reflections	

### Refinement

**Table:**

Refinement on *F*^2^	Secondary atom site location: difference Fourier map
Least-squares matrix: full	Hydrogen site location: inferred from neighbouring sites
*R*[*F*^2^ > 2σ(*F*^2^)] = 0.030	H atoms treated by a mixture of independent and constrained refinement
*wR*(*F*^2^) = 0.070	*w* = 1/[σ^2^(*F*_o_^2^) + (0.0105*P*)^2^ + 0.0726*P*] where *P* = (*F*_o_^2^ + 2*F*_c_^2^)/3
*S* = 1.04	(Δ/σ)_max_ = 0.001
4684 reflections	Δρ_max_ = 0.90 e Å^−^^3^
105 parameters	Δρ_min_ = −1.49 e Å^−^^3^
6 restraints	Extinction correction: *SHELXL97* (Sheldrick, 2008), Fc^*^=kFc[1+0.001xFc^2^λ^3^/sin(2θ)]^-1/4^
Primary atom site location: structure-invariant direct methods	Extinction coefficient: 0.242 (7)

### Special details

**Table:**

Experimental. Data were corrected for Lorentz-polarization effects and an analytical absorption correction (de Meulenaer & Tompa, 1965) was applied. The structure was solved in the P -1 space group by the direct methods (Cd and S) and subsequent difference Fourier syntheses (all other atoms), with an exception for H atoms bonded to C and N atoms which are positioned geometrically.
Geometry. All e.s.d.'s (except the e.s.d. in the dihedral angle between two l.s. planes) are estimated using the full covariance matrix. The cell e.s.d.'s are taken into account individually in the estimation of e.s.d.'s in distances, angles and torsion angles; correlations between e.s.d.'s in cell parameters are only used when they are defined by crystal symmetry. An approximate (isotropic) treatment of cell e.s.d.'s is used for estimating e.s.d.'s involving l.s. planes.
Refinement. Refinement of *F*^2^ against ALL reflections. The weighted *R*-factor *wR* and goodness of fit *S* are based on *F*^2^, conventional *R*-factors *R* are based on *F*, with *F* set to zero for negative *F*^2^. The threshold expression of *F*^2^ > σ(*F*^2^) is used only for calculating *R*-factors(gt) *etc*. and is not relevant to the choice of reflections for refinement. *R*-factors based on *F*^2^ are statistically about twice as large as those based on *F*, and *R*- factors based on ALL data will be even larger.

### Fractional atomic coordinates and isotropic or equivalent isotropic displacement parameters (Å^2^)

**Table:**

	*x*	*y*	*z*	*U*_iso_*/*U*_eq_	
Cd	0.5000	0.5000	1.0000	0.01899 (4)	
OW1	0.5857 (3)	0.24405 (19)	1.22634 (18)	0.0483 (4)	
OW2	0.77475 (16)	0.43008 (19)	0.74717 (15)	0.0293 (2)	
S	0.69724 (4)	0.72895 (4)	1.24802 (4)	0.01654 (5)	
O1	0.81539 (17)	0.55801 (17)	1.34097 (15)	0.02945 (19)	
O2	0.49364 (16)	0.76868 (19)	1.38281 (14)	0.0302 (2)	
O3	0.81079 (17)	0.89591 (15)	1.18806 (15)	0.02517 (18)	
O4	0.66952 (18)	0.69978 (16)	1.06591 (13)	0.0285 (2)	
N	0.83624 (18)	0.99858 (18)	0.75717 (15)	0.02352 (18)	
H0A	0.7789	0.9054	0.8533	0.035*	
H0B	0.7386	1.0923	0.7270	0.035*	
H0C	0.9219	1.0448	0.7964	0.035*	
C	0.9515 (2)	0.92081 (19)	0.58209 (17)	0.0226 (2)	
H1D	0.8591	0.8685	0.5400	0.027*	
H1E	1.0575	0.8180	0.6145	0.027*	
H21	0.765 (3)	0.456 (4)	0.633 (2)	0.037 (6)*	
H12	0.661 (4)	0.140 (3)	1.205 (4)	0.066 (9)*	
H11	0.561 (4)	0.235 (4)	1.347 (2)	0.045 (7)*	
H22	0.896 (3)	0.439 (4)	0.740 (3)	0.044 (6)*	

### Atomic displacement parameters (Å^2^)

**Table:**

	*U*^11^	*U*^22^	*U*^33^	*U*^12^	*U*^13^	*U*^23^
Cd	0.02244 (6)	0.01866 (6)	0.01811 (6)	−0.00551 (4)	−0.00646 (4)	−0.00462 (4)
OW1	0.0878 (12)	0.0283 (6)	0.0277 (5)	0.0176 (7)	−0.0281 (6)	−0.0090 (4)
OW2	0.0206 (4)	0.0431 (6)	0.0245 (4)	−0.0046 (4)	−0.0047 (3)	−0.0096 (4)
S	0.01696 (11)	0.01998 (12)	0.01439 (10)	−0.00478 (9)	−0.00561 (8)	−0.00337 (8)
O1	0.0256 (5)	0.0265 (5)	0.0308 (4)	−0.0015 (4)	−0.0096 (4)	0.0031 (4)
O2	0.0200 (4)	0.0437 (6)	0.0221 (4)	−0.0006 (4)	−0.0016 (3)	−0.0069 (4)
O3	0.0289 (5)	0.0231 (4)	0.0292 (4)	−0.0102 (4)	−0.0118 (3)	−0.0056 (3)
O4	0.0410 (6)	0.0350 (5)	0.0168 (3)	−0.0214 (5)	−0.0087 (3)	−0.0045 (3)
N	0.0244 (5)	0.0274 (5)	0.0175 (4)	−0.0054 (4)	−0.0040 (3)	−0.0036 (3)
C	0.0275 (5)	0.0220 (5)	0.0178 (4)	−0.0080 (4)	−0.0036 (4)	−0.0032 (4)

### Geometric parameters (Å, °)

**Table:**

Cd—OW1^i^	2.2511 (12)	S—O2	1.4708 (10)
Cd—OW1	2.2511 (12)	S—O3	1.4770 (10)
Cd—O4	2.2789 (9)	S—O4	1.4884 (8)
Cd—O4^i^	2.2789 (9)	N—C	1.4803 (16)
Cd—OW2^i^	2.2887 (10)	N—H0A	0.8900
Cd—OW2	2.2887 (10)	N—H0B	0.8900
OW1—H12	0.846 (16)	N—H0C	0.8900
OW1—H11	0.833 (15)	C—C^ii^	1.514 (2)
OW2—H21	0.833 (15)	C—H1D	0.9700
OW2—H22	0.842 (15)	C—H1E	0.9700
S—O1	1.4669 (11)		
			
OW1^i^—Cd—OW1	180.0	H21—OW2—H22	107.2 (19)
OW1^i^—Cd—O4	85.76 (5)	O1—S—O2	110.44 (7)
OW1—Cd—O4	94.24 (5)	O1—S—O3	110.09 (7)
OW1^i^—Cd—O4^i^	94.24 (5)	O2—S—O3	110.47 (7)
OW1—Cd—O4^i^	85.76 (5)	O1—S—O4	110.31 (7)
O4—Cd—O4^i^	180.0	O2—S—O4	108.81 (6)
OW1^i^—Cd—OW2^i^	95.02 (5)	O3—S—O4	106.63 (6)
OW1—Cd—OW2^i^	84.98 (5)	S—O4—Cd	134.68 (6)
O4—Cd—OW2^i^	87.96 (4)	C—N—H0A	109.5
O4^i^—Cd—OW2^i^	92.04 (4)	C—N—H0B	109.5
OW1^i^—Cd—OW2	84.98 (5)	H0A—N—H0B	109.5
OW1—Cd—OW2	95.02 (5)	C—N—H0C	109.5
O4—Cd—OW2	92.04 (4)	H0A—N—H0C	109.5
O4^i^—Cd—OW2	87.96 (4)	H0B—N—H0C	109.5
OW2^i^—Cd—OW2	180.000 (1)	N—C—C^ii^	109.62 (13)
Cd—OW1—H12	127.0 (18)	N—C—H1D	109.7
Cd—OW1—H11	127.6 (18)	C^ii^—C—H1D	109.7
H12—OW1—H11	105 (2)	N—C—H1E	109.7
Cd—OW2—H21	121.2 (16)	C^ii^—C—H1E	109.7
Cd—OW2—H22	123.0 (15)	H1D—C—H1E	108.2

Symmetry codes: (i) −*x*+1, −*y*+1, −*z*+2; (ii) −*x*+2, −*y*+2, −*z*+1.

### Hydrogen-bond geometry (Å, °)

**Table:**

*D*—H···*A*	*D*—H	H···*A*	*D*···*A*	*D*—H···*A*
N—H0A···O4	0.89	1.93	2.8155 (15)	177.
N—H0B···O2^iii^	0.89	1.99	2.8378 (16)	160.
N—H0C···O3^iv^	0.89	2.03	2.8767 (15)	160.
OW1—H11···O2^v^	0.83 (2)	1.90 (2)	2.7342 (15)	176 (3)
OW1—H12···O3^vi^	0.85 (2)	1.89 (2)	2.7293 (17)	173 (3)
OW2—H21···O1^vii^	0.83 (2)	2.01 (2)	2.8177 (14)	163 (2)
OW2—H22···O1^viii^	0.84 (2)	1.89 (2)	2.7150 (15)	165 (2)

Symmetry codes: (iii) −*x*+1, −*y*+2, −*z*+2; (iv) −*x*+2, −*y*+2, −*z*+2; (v) −*x*+1, −*y*+1, −*z*+3; (vi) *x*, *y*−1, *z*; (vii) *x*, *y*, *z*−1; (viii) −*x*+2, −*y*+1, −*z*+2.
